# Assessing the cytotoxicity of phenolic and terpene fractions extracted from Iraqi *Prunus arabica* against AMJ13 and SK-GT-4 human cancer cell lines

**DOI:** 10.12688/f1000research.131336.3

**Published:** 2024-09-10

**Authors:** Matin Adil Mahmood, Abdulkareem Hameed Abd, Enas Jawad Kadhim

**Affiliations:** 1 Department of Pharmacology, College of Medicine, Al-Nahrain University, Kadhimiya, Baghdad, Iraq; 2 Department of Pharmacology, College of Pharmacy, Al-Kitab University, Altun Kopre, Kirkuk, Iraq; 3 Department of Pharmacognosy and Medicinal Plants, College of Pharmacy, University of Baghdad, Baghdad, Iraq

**Keywords:** Prunus arabica, Phenolic, Terpene, Cytotoxicity assay, HPLC, Chou-Talalay

## Abstract

**Background:** Breast and esophageal cancer are the most aggressive and prominent causes of death worldwide. In addition, these cancers showed resistance to current chemotherapy regimens with limited success rates and fatal outcomes. Recently many studies reported the significant cytotoxic effects of phenolic and terpene fractions extracted from various *Prunus* species against different cancer cell lines. As a result, it has a good chance to be tested as a complement or replacement for standard chemotherapies.

**Methods:** The study aimed to evaluate the cytotoxicity of phenolic and terpene fractions extracted from Iraqi *Prunus arabica* on breast (AMJ13) and esophageal (SK-GT-4) cancer cell lines by using the MTT assay (3-(4,5-dimethylthiazol-2-yl)-2,5-diphenyl-2H-tetrazolium bromide). Analysis using the Chou-Talalay method was performed to assess the synergistic effect between the extracted fractions and chemotherapeutic agent (docetaxel). Moreover, high-performance liquid chromatography (HPLC) analysis was conducted for the quantitative determination of different bioactive molecules of both phenolic and terpene fractions in the extract.

**Results:** According to the findings, the treatment modalities significantly decreased cancer cell viability of AMJ13 and SK-GT-4 and had insignificant cytotoxicity on the normal cells (normal human fibroblast cell line) (all less than 50% cytotoxicity). Analysis with Chou-Talalay showed a strong synergism with docetaxel on both cancer cell lines (higher cytotoxicity even in low concentrations) and failed to induce cytotoxicity on the normal cells. Important flavonoid glycosides and terpenoids were detected by HPLC, in particularly, ferulic acid, catechin, chlorogenic acid, β-sitosterol, and campesterol.

**Conclusions:** In conclusion, the extracted fractions selectively inhibited the proliferation of both cancer cell lines and showed minimal cytotoxicity on normal cells. These fractions could be naturally derived drugs for treating breast and esophageal cancers.

## Introduction

A critical global health issue is breast cancer, the most prevalent cancer diagnosed worldwide, with 2.26 million incidents expected and it is the major cause of cancer-related deaths among women. ^1^ Although breast cancer is considered a common disease in developed societies, in 2020, the world's less developed regions accounted for two-thirds of breast cancer-related deaths and more than 50% of all breast cancer diagnoses. ^2^

In terms of cancer-related deaths, esophageal cancer (EC) occurs sixth on the list of the deadliest diseases (436,000 fatalities). Meanwhile, 473,000 cases have been recorded worldwide. ^3^ Even though chemotherapeutic regimens and radiation therapy are more effective methods for treating cancer, they are nonselective, have substantial side effects, and can harm normal healthy tissues, leading to severe unanticipated and undesirable side effects. ^4^ Initially, several tumors appeared amenable to treatment. However, with time, resistance might develop for a number of reasons, such as mutations in DNA and metabolic changes that cause the drug to be inhibited and degraded. ^5^

The occurrence of natural compounds as anticancer agents is estimated to exceed 60% of the current anticancer drugs. ^6^ These drugs from natural origins can be used for both cancer prevention and treatment due to their pharmacological safety and can be used independently or in conjunction with existing chemotherapeutic treatments to improve the therapeutic efficacy or to reduce chemotherapy-induced toxic effects. ^7^ The constant change and injury that occurs in the human body over decades requires a development of revolutionary and highly precise “arms” capable of successfully combating malignant cells. Natural products are a powerful and endless source for identifying the finest anticancer prospects. ^8^

*Prunus arabica* is recognized as a distinct species from the farmed almond *P.* *dulcis.* Both, however, are members of the *Prunus* genus and the *Rosacea* family. ^9^ This species was given its scientific name based on its geographical location where it first appeared. This taxon is indigenous to mild climate Asia regions, including the Fertile Crescent Mountains, as well as Turkey, Iran, and Iraq. ^10^

*Prunus arabica* is a thick undomesticated almond species with an unbarked stem that remains green even during dormancy. ^9^ A wide range of biological and pharmacological effects from different *Prunus* species show great promise for the treatment of various cancers. ^11^ Flavonoids, steroids, terpenoids, poly phenols, and other chemicals have all been identified from various *Prunus* species. ^12^ Polyphenols and terpenoids which are found plentifully in plants, show anticancer effects, via inhibiting cancer cell growth, blood vessel formation, metastasis, inflammation, and inducing cell death. ^13^ For this purpose, the anticancer effect of phenolic and terpene fractions from *Prunus arabica* extract was investigated using the AMJ13 and SK-GT-4 cancer cell lines and normal human fibroblasts (NHF).

## Methods

### Preparation and separation of plant extract

In March 2021, whole *Prunus arabica* plants were collected from North of Erbil. Dr. Sukaena Abbas, Department of Biology at the College of Science, University of Baghdad, identified and authenticated the plant. All plant parts were washed, dried in the air for 12 days, including roots, stems, thorns, leaves, and flowers. An electric grinder was used to crush and grind the dried material (leaves) into a course powder. The voucher number was assigned to the plant (UBPH-2021-18) and voucher specimens deposited in the herbarium of the Department of Pharmacognosy and Natural Medicine, University of Baghdad, Iraq. The full protocol can be found in the *Extended data.* ^50^ Approximately 500 gm of granular powdered plant leaves that had been shade-dried for 12 days then defatted for 24 hours with hexane (BDH chemicals, England cat-no. BDH24575.100E) in a ratio of 1:3 W/V before being dried at room temperature. In a Soxhlet apparatus (BOECO, Germany) the defatted plant components were separated using two liters of 80% ethanol (Sigma-Aldrich, Germany cat-no1070172511) until completely depleted. A thick, dark-greenish-yellow residual (known as the crude fraction A) was obtained by drying the alcoholic extract by evaporation at low pressure and temperatures below 40°C by using IKA RV 10 Rotary Evaporator (Germany). This fraction was acidified using 300 ml of 5% HCl (Sigma-Aldrich cat-no. 1009861000) to pH 2 and then split with ethyl acetate (Sigma-Aldrich, Germany cat-no. 319902-1L) to acquire two distinct layers (the acidic aqueous layer and ethyl acetate layer-crude fraction). The crude fraction was dried out using low-pressure evaporation in an IKA RV 10 Rotary Evaporator (Germany) then basified with 300 ml of 5% NaOH (Honeywell, USA cat-no. 30620) to pH 10 and extracted by adding chloroform (Honeywell, USA cat-no. C2432) to the separatory funnel in order to obtain two layers; the aqueous basic layer and the and chloroform layer. The basic water layer evaporated to the point of dryness and acidified with 5% HCl to reach pH 2, then extracted with ethyl acetate to get another fraction designated as fraction (F-B). The chloroform layer which was separated by the same steps and partitioned with 80% methanol (Biochem Chemopharma, France cat-no. 213032500) and petroleum ether (Sigma-Aldrich, Germany cat-no. 32299) to obtain another two layers; fraction (C) the petroleum ether and methanol fraction which was considered as fraction D. ^14^

### Preliminary qualitative phytochemical analysis of fractionated extract of *Prunus arabica*

The full protocol can be found in the *Extended data.* ^51^ Standard protocols were used in chemical testing to identify the active components using ethanolic extracts from various plant fractions. ^15^

I.
**Alkaloids test** : Precisely 2 ml of alcoholic extract and fractions were stirred with 5 ml of 1% HCl (Sigma-Aldrich cat-no. 1009861000) on a steam bath. Mayer’s reagent (prepared by dissolving 1.35 gm mercuric chloride (Sigma-Aldrich, Germany ca-no. 215465) in 60 ml water + 5 gm potassium iodide (Sigma-Aldrich, Germany cat-no. 221945) in 10 ml water) and Wagner’s reagent (prepared by dissolving 1.27 gm of iodine (Sigma-Aldrich, Germany cat-no. 1047630050) and 2 gm of potassium iodide in 100 ml of water) were used. White and reddish-brown colored precipitates were considered as indications of the presence of alkaloids.
II.
**Flavonoids tests**

  a. Lead acetate test: precisely 1 ml of 10% lead acetate solution (BDH limited, England cat-no. LL0093) was incorporated into 2 ml of alcoholic extract and fractions. The presence of a yellowish-white precipitate indicated the presence of flavonoids.
  b. NaOH test: 2 ml of the extract and fractions were subjected to aqueous NaOH and HCl; the development of a yellowish-orange color indicated the presence of flavonoids.
III.
**Steroids tests**

  a. H _2_SO _4_ test: 2 ml of sulfuric acid (BDH limited, England cat-no. BDH3068-500MLP) was added to the extract, a green color was formed as an indication to the presence of steroids.
IV.
**Terpenoids test (Salkowski test):** 5 ml of plant extract mixed with 2 ml of chloroform (Honeywell, USA cat-no. C2432), and 3 ml of concentrated sulphuric acid (BDH limited, England cat-no. BDH3068-500MLP) was carefully added to form a layer. A reddish-brown coloration of the inter face was formed to show positive results for the presence of terpenoids.

### Ultrasonic Extraction of Phenolic Compounds and terpenes

**Reference standards and reagents**

The reference standards (Caffeic acid cat-no C0625, (+)-Catechin hydrate cat-no. C1251, Chlorogenic acid cat-no. 00500590, Ferulic Acid cat-no. PHR1791, Gallic acid monohydrate cat-no. 27645, p-Coumaric acid cat-no. C9008, Quercetin cat-no. Q4951, Rutin hydrate cat-no. R5143, β-Sitosterol cat-no. 43623, Campesterol cat-no. C5157, Stigmasterol cat-no. 47132) were purchased from Sigma Aldrich, Germany. The pure water used in the study was distilled with a Milli-Q. (Millipore, Bedford, MA, USA). Chemicals, including methanol (cat-no. 106007), acetonitrile (cat-no. 100030), and acetic acid (cat-no. 543808) (HPLC grade), were all ordered from Merck Ltd, Mumbai, India. Before usage, the solvents were processed using 0.45 mm pore size (Millipore) membrane filters.

**Instrumentation and analytical conditions**

Individual phenolic components identification was conducted by reversed-phase high-performance liquid chromatography utilizing a Sykam HPLC chromatographic system (Germany) integrated with ultraviolet detection (Sykam S 3240 UV/Vis Multichannel Detector, Germany) and sample delivery system (Sykam S1122 Solvent deliver system, Germany). The column temperature was maintained at 30 °C (Sykam S 4011 column thermo controller, Germany). The gradient elution method, with eluent A and eluent B (methanol and 1% (v/v) formic acid in water respectively), was carried out as follows: initial 0–4 min, 40% B; 4–10 min, 50% B; and flow-rate of 0.7 ml/min. Approximately 100 μL of the samples were injected. An autosampler (Skynm S5200 sample injector, Germany) analyzed the standards automatically. Spectral data was recorded at a 280 nm. ^16^

The following conditions were used for the terpene fraction; mobile phase acetonitrile: distilled water: acetic acid (60:25:5), column = C18-ODS (25 cm * 4.6 mm), detector = UV- 220 nm, and the flow rate was 1 ml/min.

### Cell lines

AMJ13 breast cancer ^17^ and NHF normal cell lines (normal human derived adipose tissue) ^18^ were cultivated in Roswell Park Memorial Institute medium RPMI 1640 (Capricorn, Germany cat-no. RPMI-A) with 10 % fetal bovine serum (FBS) (Capricorn, Germany cat-no. FBS-22A), antibiotics (penicillin and streptomycin) (Capricorn, Germany cat-no. PS-B) then incubated at 37 °C. SK-GT-4, the esophageal cancer cell line, ^19^ was maintained in minimum essential medium MEM (Capricorn, Germany cat-no. MEMA-RXA). Cells were passaged using Trypsin-EDTA (Capricorn, Germany cat-no. TRY-1B), replanted at 50% confluence twice weekly, and incubated at 37 °C (Cypress Diagnostics, Belgium). ^17^

AMJ13, NHF cell lines provided by the Iraqi Center for Cancer Research and Medical Genetics. SK-GT-4 were supplied by European Collection of Authenticated Cell Cultures (ECACC).

### Cytotoxicity assays

The full protocol can be found in the *Extended data.* ^52^ The MTT assay for cell viability ^20^ was performed to measure the cytotoxic effect of the extracted fractions. Cell lines were planted into 96-well plates (Santa Cruz Biotechnology, USA cat-no. sc-204447) at 1 × 10 ^4^ cells/well. After 24 hrs, or until a confluent monolayer was achieved, cells were treated with the tested compound. After 72 hours of exposure, cell viability was assessed; the medium was removed by aspiration and 28 μL of 2 mg/ml MTT (MTT stain obtained from Bio-World, USA cat-no. 42000092-1) was added and incubated at 37 °C for 1.5 hours.

After removing the MTT solution, the crystals remaining in the wells were solubilized by the addition of 130 μL of dimethyl sulfoxide (DMSO) (Santacruz Biotechnology, USA cat-no. sc-202581) was used to solubilize the residual crystals in the wells, proceeded by incubation for 15 min at 37 °C with shaking. ^21^ Using a microplate reader (Genex-lab, USA cat-no. MR-100) the absorbance was measured at 492 nm; the experiment was carried out in triplicate. Untreated cells, MTT solution, and a solubilizing buffer (DMSO) will be placed in the wells designated as a control. Due to its proven lack of cytotoxicity in cell culture, a final concentration of 0.5% DMSO was used for this study. The following formula was applied to determine the percent of cytotoxicity ^22^:

$$\%\text{Cell viability}=\left(\frac{\mathrm{AT}}{\mathrm{ANT}}\right)\times 100$$

%Cytotoxicity=100−cell viability

AT: Absorbance of treated cells with tested compounds, ANT: absorbance of untreated cells.

### Chou-Talalay analysis

Synergism or interaction of phenolic and terpene fractions with docetaxel was investigated using a non-constant ratio. Analysis of the combination was performed by the Chou-Talalay method. CompuSyn was used to derive the corresponding combination indices (CI) (CompuSyn, Inc., Paramus, NJ, USA). The combination indices were calculated using non-constant phenolic, terpene, and docetaxel ratios and mutually exclusive formulations. A CI value between 0.9 and 1.1 indicates an additive effect, a CI value below 0.9 denotes synergy, and a CI above 1.1 denotes antagonism. ^23^

### Selectivity Index analysis

The Selectivity Index (SI) is calculated by dividing a compound’s IC _50_ value against normal cells by its IC _50_ value against cancer cells. ^24^^,^^25^ High-selectivity compounds have a SI value greater than 3, while low-selectivity compounds have a SI value of 3. ^25^

### Statistical analysis

The MTT assay results were analyzed statistically with an ANOVA test in GraphPad V 7.00 (for windows), an open-access alternative that can perform an equivalent function is R. The unpaired t-test was used to compare groups; P-values <0.05 were considered statistically significant.

## Results

### Qualitative phytochemical analysis

The phytochemical screening results are given in Table 1.

**Table 1.** Phytochemical analysis of *Prunus arabica* crude extract and isolated fractions.

Crude and fractions	Alkaloids	Flavonoids	Steroids	Terpenoids
Crude A	+	+	+	+
Fraction B	-	+	-	+
Fraction C	-	-	-	+
Fraction D	-	-	+	+

+, - represent the presence and absence of phytoconstituents, respectively.

To discover individual compounds of extracted fractions, HPLC analysis was used. Seven phenolic acids (p-coumaric acid, ferulic acid, gallic acid, caffeic acid, quercetin, rutin, catechin) and three terpenes (B sitosterol, campesterol, stigmasterol) were detected from extracted fractions of *Prunus arabica*, as shown in Tables 2 and 3.

**Table 2.** Phenolic acid and corresponding concentration expressed in mg/gm of dry fraction extract.

Phenolic acid	Retention time in min	Conc. mg/gm
P-coumaric acid	2.18	1.954
Ferulic acid	3.22	3.992
Gallic acid	4.79	1.618
Caffeic acid	6.08	1.925
Quercetin	7.89	2.125
Rutin	8.33	1.328
Catechin	10.2	2.451
Chlorogenic acid	12.08	2.491
Unknown	14.05	–

**Table 3.** Terpenes and corresponding concentration expressed in mg/gm of dry fraction extract.

Terpenes	Retention time in min	Conc. mg/gm
Unknown	2.69	–
B sitosterol	4	3.979
Unknown	4.79	–
Campesterol	11.5	4.358
Stigmasterol	10	2.350

### *In vitro* cytotoxic activity

**Figure 1.**
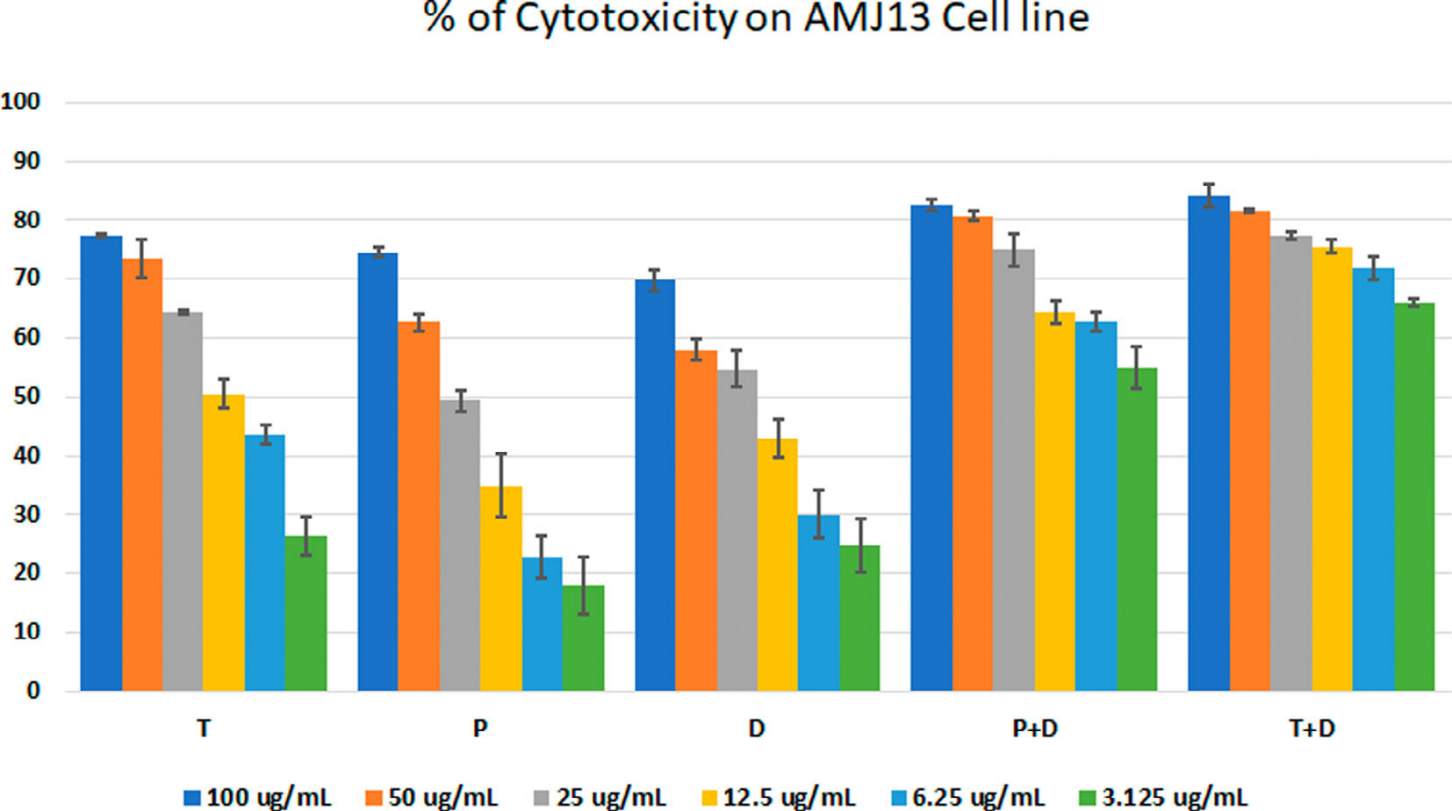
The cytotoxicity of different concentrations on the AMJ13 cell line, T: terpenes, P: phenolic, D: docetaxel, P+D: phenolic and docetaxel, T+D: terpene and docetaxel.

**Figure 2.**
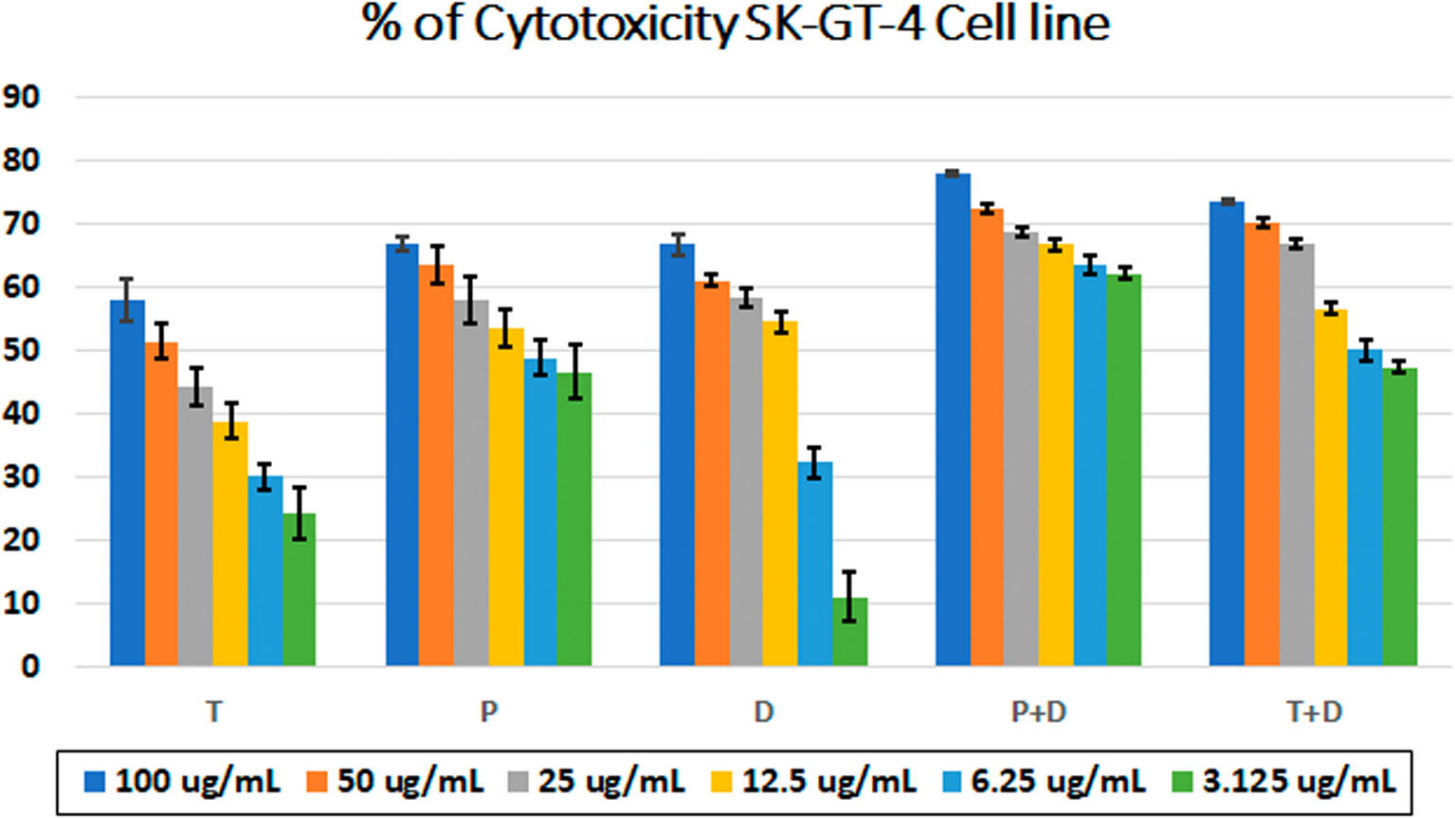
The cytotoxicity of different concentrations on the SK-GT-4 cell line, T: terpenes, P: phenolic, D: docetaxel, P+D: phenolic and docetaxel, T+D: terpene and docetaxel.

**Figure 3.**
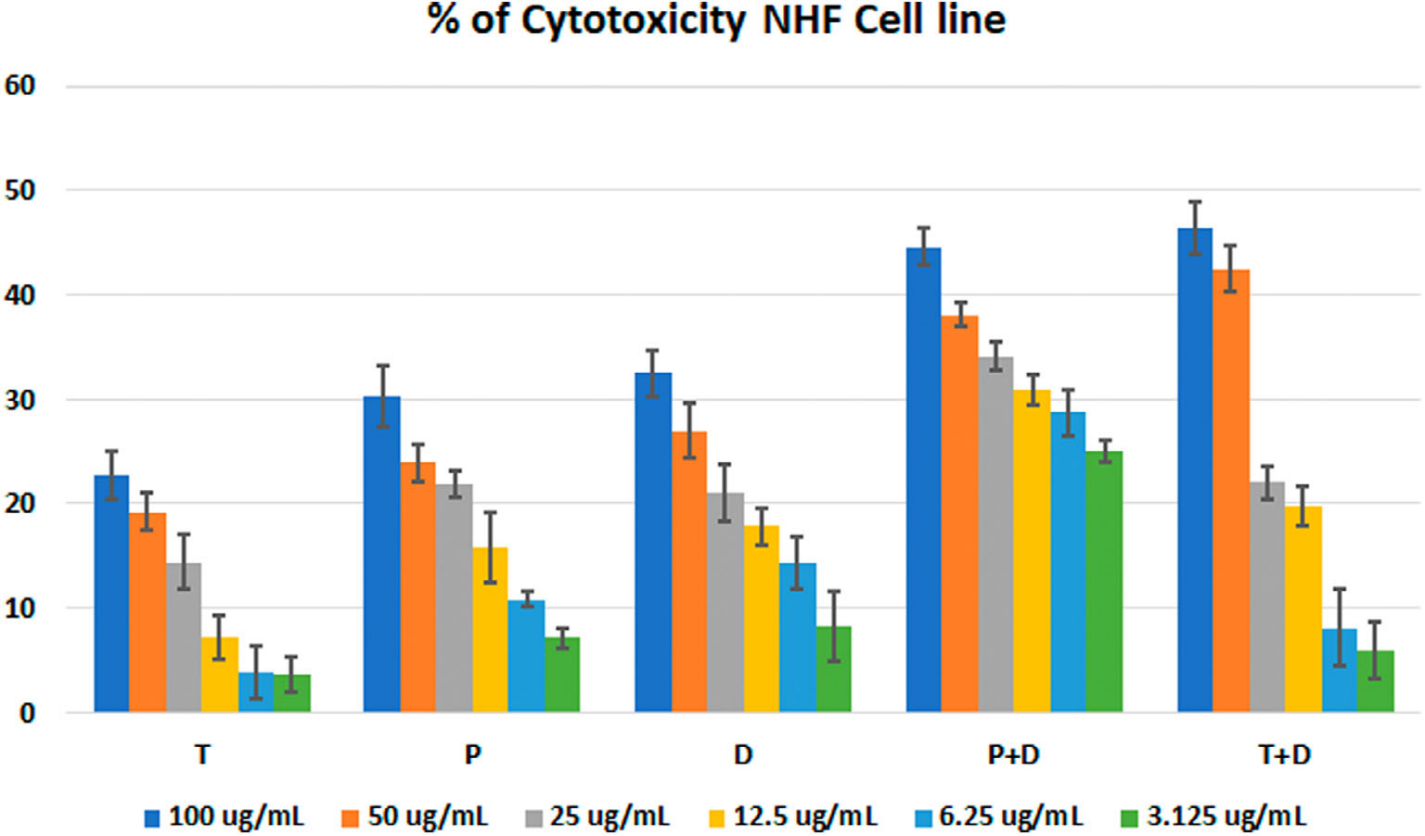
The cytotoxicity of different concentrations on the NHF cell line, T: terpenes, P: phenolic, D: docetaxel, P+D: phenolic and docetaxel, T+D: terpene and docetaxel.

**Figure 4.**
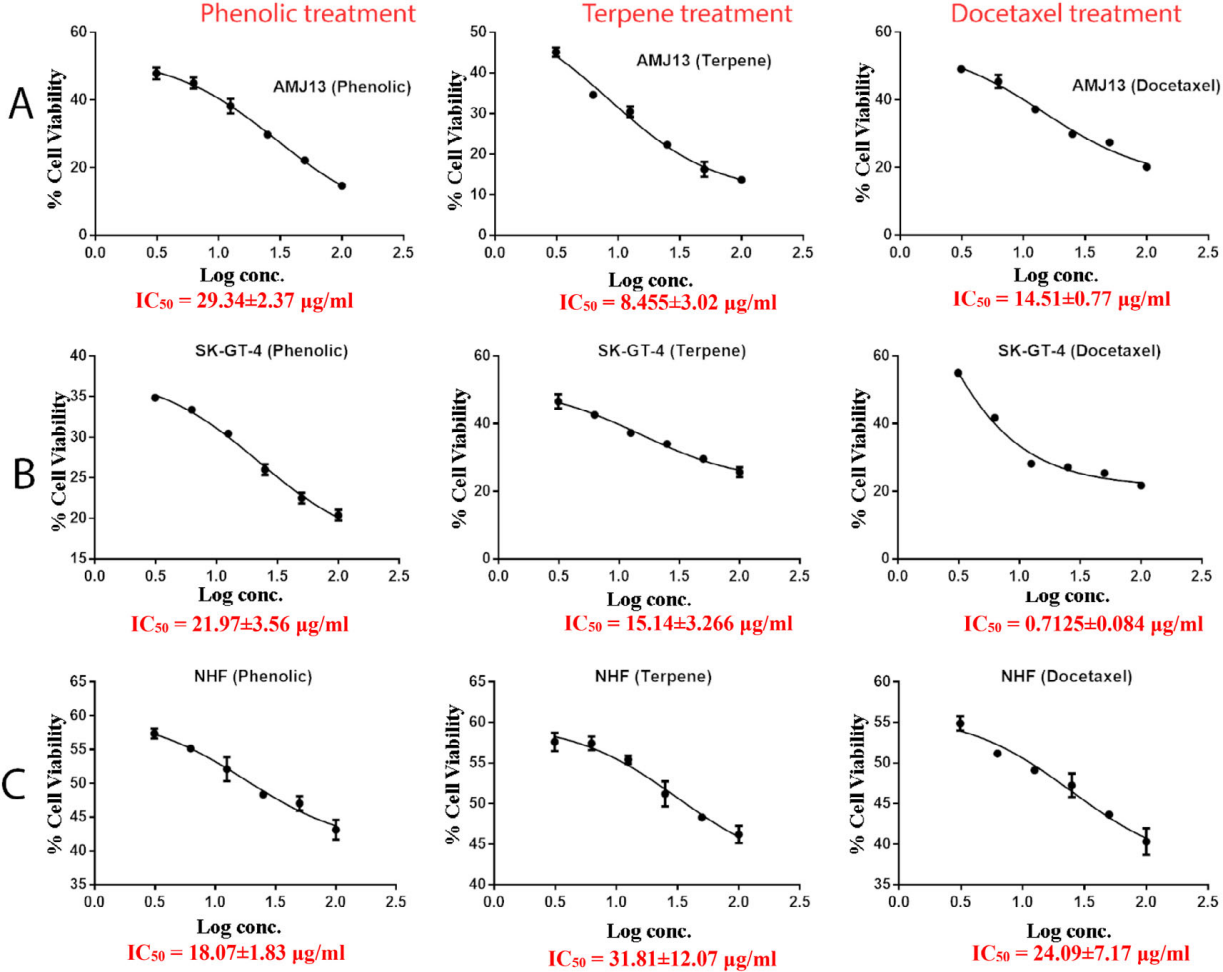
The half maximal inhibition concentration (IC _50_) for phenolic, terpene, and docetaxel, respectively, on A: AMJ13, B: SK-GT-4, and C: NHF cell lines.

The MTT assay was used to evaluate the cytotoxicity and therapeutic efficacy of the isolated fractions in the cancer and normal cell lines (AMJ13, SK-GT-4 and NHF). As shown in Figures 1– 3, the results showed that the treatments significantly decreased the viability of the cancer cells (>50% at higher concentrations) with minimal cytotoxic effects on the normal cells (<50% cytotoxicity). On the AMJ13 cell line, phenolic, terpene fractions, and docetaxel had a half maximal inhibitory concentration (IC _50_) of 29.34±2.37, 8.455±3.02, and 14.51±0.77 μg/ml, respectively, as shown in Figure 4. On the SK-GT-4 cell line, phenolic, terpene fractions, and docetaxel had an IC _50_ of 21.97±3.56, 15.14±3.266, and 0.7125±0.084 μg/ml, respectively, as shown in Figure 4. While on the NHF cell line, the phenolic, terpene, and docetaxel had an IC _50_ of 18.07±1.83, 31.81±12.07, and 24.9±7.17 μg/ml, respectively.

### Extract-docetaxel potential interaction (Chou–Talalay analysis)

**Figure 5.**
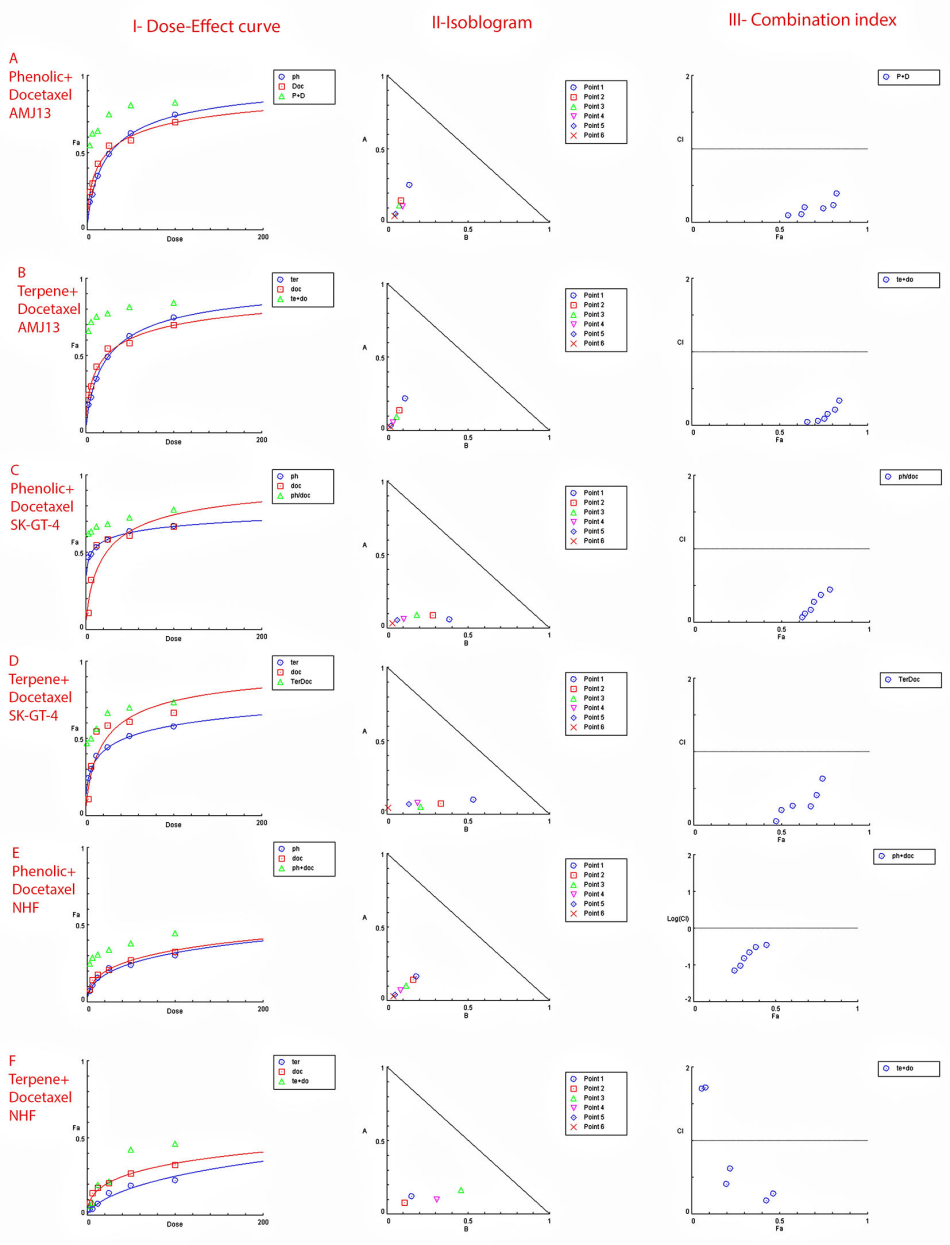
The dose–response curves, the normalized isobologram and combination index with non-constant ratios. Data is expressed as fraction affected (fa) against combination index plots. Combination index (CI) value less than 0.9 indicates synergy, CI values between 0.9 and 1.1 indicates additive, and CI value larger than 1.1 indicates an antagonism. (A and B) Represents the effect against AMJ13 cell line by phenolic-docetaxel and terpene-docetaxel combinations respectively, all points are showing synergy to very strong synergy; (C and D) represents the effect against SK-GT-4 cell line by phenolic-docetaxel and terpene-docetaxel combinations respectively, all of the points are showing synergy to very strong. (E and F) Show the effect on the NHF cell line by phenolic-docetaxel and terpene-docetaxel combinations respectively, tested points showed inconsiderable cytotoxicity as all concentrations failed to emerge 50% cytotoxicity. The data represents six separate experiments.

The possible interactions between extracted fractions (phenolic, terpene) and docetaxel therapy on the AMJ13 and SK-GT-4 cell lines were analyzed. The quantification of synergism or antagonism is defined as a mass-action law issue (determined by the combination index CI values) and cannot be determined by the statistical p values. ^23^ Chou-Talalay equations were used to calculate the combination's value. A CI value less than 0.9, the effects were assumed to be synergistic; a CI value between 0.9 and 1.1, the effects were considered as additive; while a value larger than 1.1, the effects were assumed to be antagonistic. ^26^ After an exposure period of 72 hours, the phenolic fraction of *P. arabica* with docetaxel produced a strong to very strong synergic cytotoxic effect against AMJ13 and SK-GT-4 cancer cell lines in comparison with a single treatment. The terpene fraction showed almost the same synergism effect when combined with docetaxel ( Figure 5).

### Morphological analysis (crystal violet staining)

**Figure 6.**
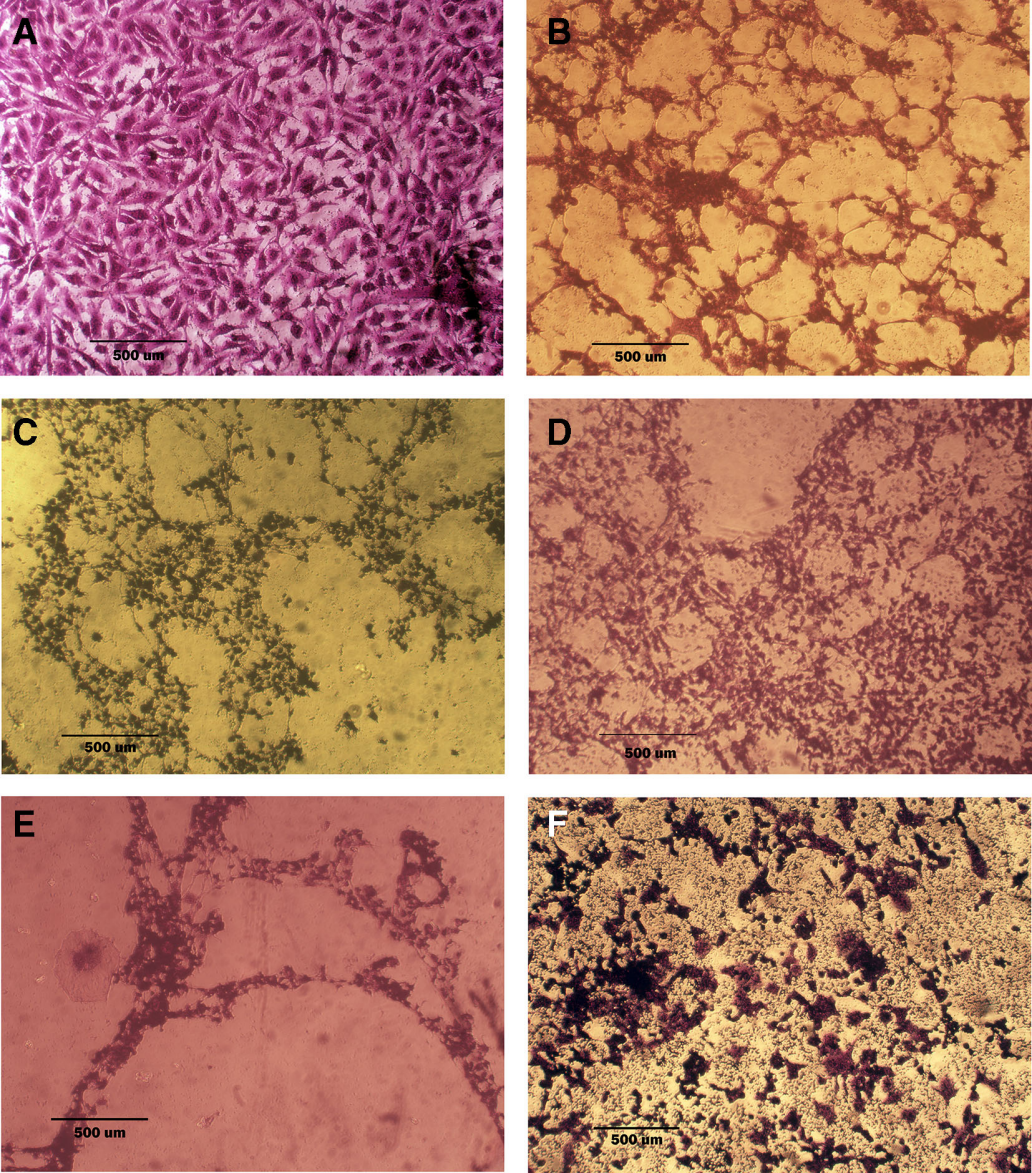
Morphology of AMJ13 cell line. A: untreated cells show multipolar elongated epithelial-like shape, with multiple nuclei in most of the cells and nuclear polymorphism, many cells showed mitotic figures B, C, and D cell treated with terpene fraction phenolic fraction and docetaxel respectively; the treated cells showed a shrinkage, cytoplasm and cell membrane disappearance, stromal edema, nucleus shrinkage and marked decrease in the number of cells. E and F cells were treated with a combination therapy of phenolic plus docetaxel and terpene plus docetaxel, respectively; the treated cells showed more prominent cytotoxic effects than single treatment with a dense nucleus. The microscopic images were captured at 10× by an inverted microscope (IXplore Standard Olympus, Japan).

**Figure 7.**
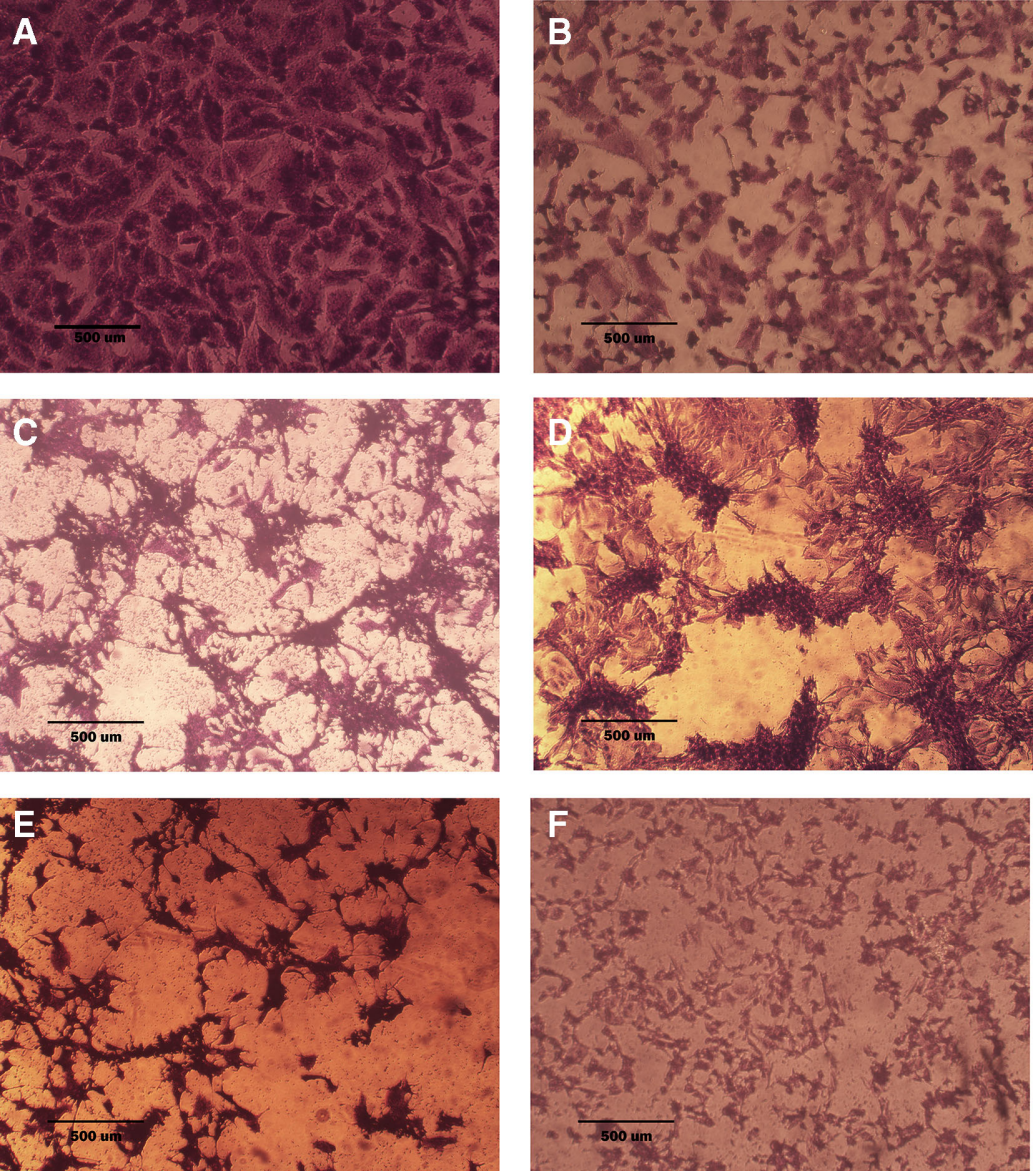
The morphology of SK-GT-4 cell line. A: untreated cells are squamous or poorly differentiated and irregular in shape. B: cells treated with terpene fraction showed shrinkage; the squamous cell border remained intact, while other cells (no bordered cells) showed stromal edema. C: cells treated with phenolic fraction showed an increment of the stromal edema and disappearance of squamous cells with no ductal nuclear aggregation. D: docetaxel-treated cells showed focal aggregation and cellular shrinkage. E and F cells were treated with combination therapy phenolic plus docetaxel and terpene plus docetaxel, respectively; the treated cells showed more shrinkage than single treatment (very small sized cells), the squamous cell border is intact, destruction of ductal/basal cell membranes with no focal aggregation. The microscopic images were captured at 10× by an inverted microscope (IXplore Standard Olympus, Japan).

**Figure 8.**
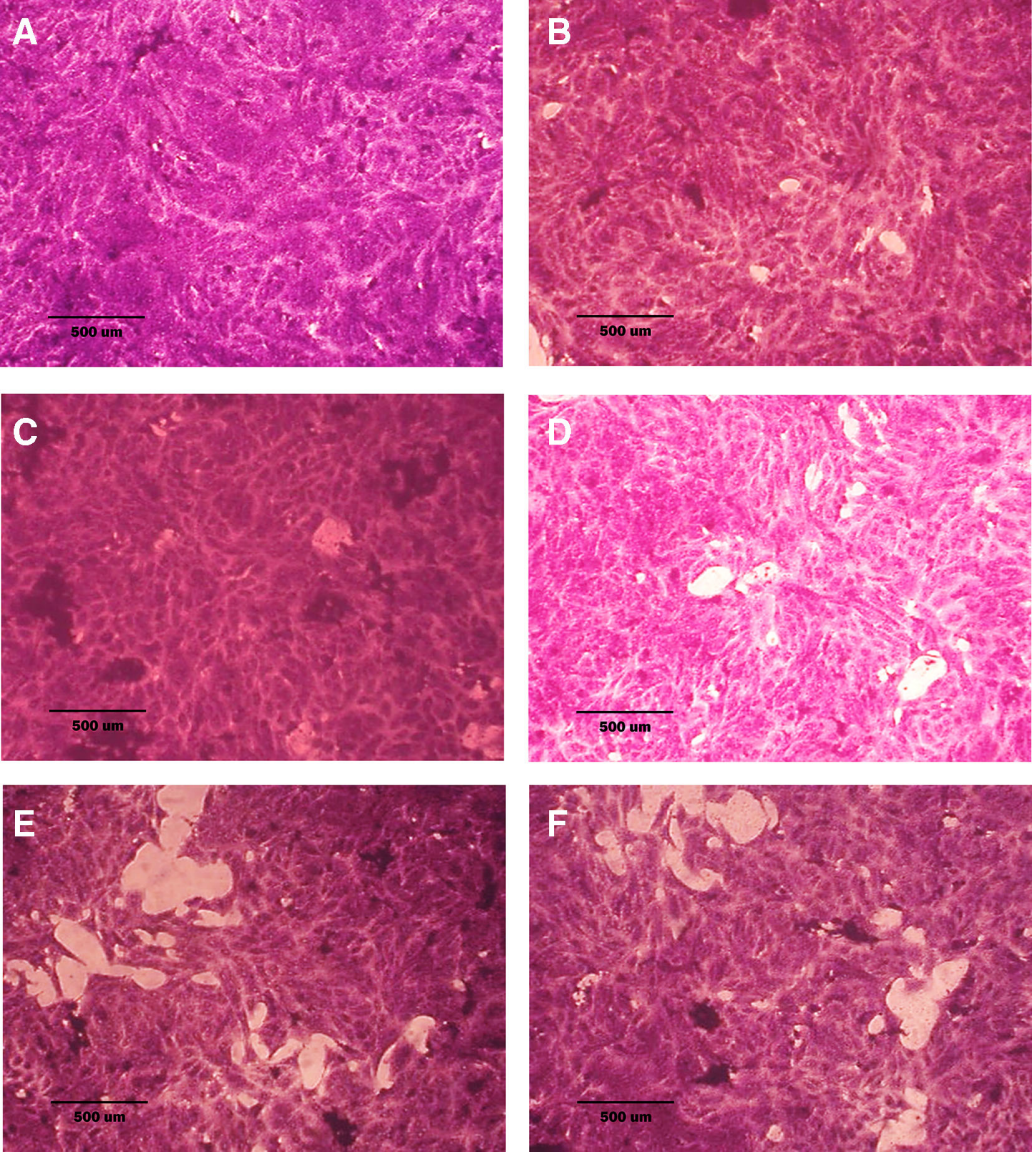
Morphology of NHF cell line. A: untreated cells; cells appear as plump spindle-shaped or stellate-shaped cells with centrally placed oval or round nuclei. B: cells treated with terpene fraction showed mild apoptosis. C: cells treated with phenolic fraction showed condensation of cells with no edema. D: docetaxel-treated cells showed focal apoptosis with mild stromal edema, and the cells remained the same size. E and F cells were treated with a combination therapy of phenolic plus docetaxel and terpene plus docetaxel, respectively; the treated cells showed prominent apoptosis compared to the single treatment. The microscopic images were captured at 10× by an inverted microscope (IXplore Standard Olympus, Japan).

Morphological alterations were examined with crystal violet staining of the cell lines after 72 hr. Exposure to the IC _50_ of the phenolic fraction, terpene, docetaxel, and their combinations with chemotherapy as shown in Figures 6, 7 and 8. The captured images are for cells treated with (100 μg/ml) of the tested fractions or their combinations with docetaxel on cancer or normal cell lines.

### Selectivity index

The provided Table 4 illustrates the selectivity index for the tested compound in this study.

**Table 4.** IC _50_ values and SI of phenolic, terpene and docetaxel treatments on cancer cell lines.

Compound	Cell line	IC _50_ μg/ml	SI
Phenolic fraction	AMJ13	29.34±2.37	0.61
	SK-GT-4	21.97±3.56	0.82
	NHF	18.07±1.83	
Terpene fraction	AMJ13	8.455±3.02	3.76
	SK-GT-4	15.14±3.26	2.10
	NHF	31.81±12.07	
Docetaxel	AMJ13	14.51±0.77	1.66
	SK-GT-4	0.7125±0.084	33.81
	NHF	24.09±7.17	

Data are means ± SD of the IC _50_ in μg/ml for each treatment, against the evaluated cell lines. The selectivity index (SI) represents IC _50_ for the normal cell line divided by the IC _50_ for the cancerous cell line after 72 h. Docetaxel was used as a positive control.

## Discussion

Polyphenols and terpenes are the most abundant and widely distributed compound in the plant kingdoms and groups. ^27^ *Prunus* species have been found to be a potential dietary supplement and a good source of phenolic and terpene bioactive chemicals. ^28^

In the current study, lead acetate and NaOH tests for polyphenols gave a positive result, meaning the presence of phenolic compounds in *P. arabica* extract. The dark color might indicate the presence of large quantities of polyphenols and flavonoids. ^29^ The H _2_SO _4_ test gave a dark pink or red color and greenish color, respectively, as an indication of the presence of steroids, while the chloroform and sulphuric acid test produced a greyish color which was considered an indication of the presence of terpenes.

The data for the HPLC analysis showed that the phenolic fraction of the extracted *P. arabica* contains eight phenolic compounds (p-Coumaric acid, ferulic acid, gallic acid, caffeic acid, quercetin, rutin, catechin, and chlorogenic acid), the terpene fraction contained three major terpenes (β-sitosterol, stigmasterol, and campesterol) as well as some non-phenolic organic and inorganic components at deceptive values.

From the eight phenolic compounds found in our phenolic fraction of the *P. arabica* extract, the highest component concentration was ferulic acid (3.992 mg/gm) with a retention time of 3.22 min, which may contribute to the high efficacy of the phenolic extract as a cytotoxic biological chemical on different cancer cell lines. ^30^ Research has indicated that ferulic acid induces cell death by decreasing the Bcl-2 and increasing the *BAX* gene expression or by upregulation of caspase-3 and cleaved caspase-9. ^31^ Moreover, the significant contribution of chlorogenic acid and catechin in manifesting anti-proliferative effects in cancer cell lines is underscored. This assertion is substantiated by research findings demonstrating that quercetin induces apoptosis in MCF-7 cells and hinders the proliferation of MCF-7 breast cancer cells in a time- and concentration-dependent manner. The inhibitory impact of quercetin on MCF-7 cell growth, coupled with its promotion of apoptosis through G0/G1 phase arrest, has been established. ^32^ Additionally, catechins exert anticancer effects by modulating various processes, encompassing the inhibition of carcinogenic activity, tumorigenesis, proliferation, apoptosis induction, cell cycle arrest, metastasis, and angiogenesis. ^33^ Rutin, as another extract component from *Prunus arabica*, was found to possesses anti-proliferative activities by triggering apoptosis in triple-negative breast cancer. ^34^

The highest component of the terpene fraction was campesterol (4.358 mg/gm), with a retention time of 11.5 min. A study by Hyocheol B. *et al.* confirmed that campesterol could inhibit both cellular growth and cell cycle progression by regulating the PCNA (proliferating cell nuclear antigen) and PI3K/MAPK (phosphatidylinositol-3-kinase/mitogen-activated protein kinase) signal pathways. Moreover, their results also showed that campesterol could prevent the clustering of ovarian cancer cells. ^35^ Some undiscovered phenolic and terpene compounds may be presented by peaks on the chromatograms, hydroperoxides or peroxides produced from terpenes are likely responsible for these found but unidentified peaks. ^36^

The present work studied the cytotoxic effects of the extracted fractions (phenolic and terpene) of *P. arabica* alone and in combination with docetaxel and compared their novel effects with the single chemotherapeutic agent (docetaxel) against AMJ13, SK-GT-4, and NHF cell lines. Breast cancer and esophageal carcinoma are considered highly malignant tumors, which lead to poor prognoses. ^37^ The low efficacy of currently available breast and esophageal cancer chemotherapeutics and radiation moreover these therapies are associated with severe adverse effects and patients can develop resistance to these agents. ^38^^,^^39^

*In vitro*, the results of this study appear that the treatment with the phenolic and terpene extract of *P. arabica* significantly reduced cell viability and triggered apoptosis when compared to the control group in both AMJ13 and SK-GT-4 cell lines ( Figures 1– 2). During this study against the AMJ13 cell line, the terpene fraction showed comparable cytotoxic effects to docetaxel even in concentrations as low as 25 μg/ml, the IC _50_ for phenolic terpene and docetaxel against AMJ13 cell line was 29.34±2.37, 8.455±3.02 and 14.51±0.77 μg/ml respectively ( Figure 4).

The phenolic fraction showed almost an equal cytotoxicity to docetaxel against the SK-GT-4 cell line, while terpene showed less significant cytotoxicity in comparison with other tested treatments; the IC _50_ for phenolic terpene and docetaxel against SK-GT-4 was 21.97±3.56, 15.14±3.26, 0.7125±0.084 μg/ml respectively as shown in Figure 4. All treatment concentrations for phenolic and terpene fractions failed to show significant cytotoxicity on the NHF cell line (less than 50%).

Many unique chemical components, including polyphenols, flavonoids, alkaloids, and terpenes, have been identified from *Prunus* species. The great structural variety of these compounds underlies their unique biological properties, which include bioavailability, antioxidant activity, and specific interactions with cell receptors and enzymes. ^40^

Researchers have found that flavonoids have a wide range of biological effects in mammals, including antimicrobial, antiviral, analgesic, anti-allergic, hepatoprotective, cytostatic, and apoptotic properties. ^41^

The current study confirms the findings of previous studies which showed that phytosterols, such as quercetin and β-sitosterol, protect against a wide variety of diseases and exhibit selective cytotoxicity towards cancer cells, as evidenced by high apoptosis indices in cells exposed to quercetin’s anticancer activity. ^42^

Many studies have demonstrated that flavonoids have cytotoxic effects, including modulating ROS-scavenging enzyme activities, cell cycle arrest, induction of apoptosis and autophagy, and suppression of cancer cell proliferation and invasiveness. In healthy cells, flavonoids function as antioxidants, but in cancer cells, they become strong pro-oxidants that induce apoptotic pathways and downregulate pro-inflammatory signals. ^43^

Stigmasterol's cytotoxic actions come from its ability to induce autophagy in tumor cells, decrease their proliferation and spread, and promote their apoptosis. ^44^

The increased sensitivity of cancer cells to cytotoxicity was another goal of this investigation by using phenolic or terpene fractions with docetaxel and the cumulative effects of many dosages. The MTT assay performed with docetaxel in the presence of varying amounts of phenolic or terpene fractions. According to the findings, the phenolic, terpene, and docetaxel combination substantially decreased cancer cell viability without causing appreciable damage to normal cells. The Chou-Talalay equation was used to evaluate the combinations.

The degree of synergy or antagonism cannot be assessed by p value in a statistical manner but can be quantified using CI values (combination index values). ^45^ Nearly all of the doses examined showed synergistic cytotoxicity against the cancer cell lines. To demonstrate their safety, testing on a normal human fibroblast cell line showed no effect at any dose of the combination of phenolic and terpene fractions with docetaxel. There have been a number of studies suggesting that phenolic acids and terpenes may boost the effect of other chemotherapies on breast cancer. ^46^ However, this is the first study to provide empirical evidence of synergy between phenolic and terpene fractions with docetaxel against AMJ13 and SK-GT-4 cell line ( Figure 5 and Table 5).

**Table 5.** AMJ13, SK-GT-4, and NHF cells were treated with phenolic fractions or terpene fractions in combination with docetaxel and after 72 hours at the indicated doses, an MTT assay was conducted.

A		AMJ13	Phenolic with Docetaxel		
Points	Ph. Conc ug/ml	Doc. Conc. ug/ml	Cytotoxicity	CI	Effect
1	50	50	82.6%	0.396	Synergism
2	25	25	80.7%	0.238	Strong Synergism
3	12.5	12.5	75.0%	0.194	Strong Synergism
4	6.25	6.25	64.3%	0.209	Strong Synergism
5	3.125	3.125	62.7%	0.116	Strong Synergism
6	1.5625	1.5625	54.9%	0.095	Very Strong Synergism

B		AMJ13	Terpene with Docetaxel		
Points	Ter Conc ug/ml	Doc. Conc. ug/ml	Cytotoxicity	CI	Effect
1	50	50	84.2%	0.334	Synergism
2	25	25	81.7%	0.216	Strong Synergism
3	12.5	12.5	77.5%	0.158	Strong Synergism
4	6.25	6.25	75.5%	0.093	Very Strong Synergism
5	3.125	3.125	71.9%	0.062	Very Strong Synergism
6	1.5625	1.5625	66.0%	0.047	Very Strong Synergism

C		SK-GT-4	Phenolic plus Docetaxel		
Points	Ph. Conc ug/ml	Doc. Conc. ug/ml	Cytotoxicity	CI	Effect
1	50	50	77.8%	0.443	Synergism
2	25	25	72.5%	0.373	Synergism
3	12.5	12.5	68.6%	0.276	Strong Synergism
4	6.25	6.25	66.7%	0.168	Strong Synergism
5	3.125	3.125	63.5%	0.119	Strong Synergism
6	1.5625	1.5625	62.1%	0.069	Very Strong Synergism

D		SK-GT-4	Terpene plus Docetaxel		
Points	Ter Conc ug/ml	Doc. Conc. ug/ml	Cytotoxicity	CI	Effect
1	50	50	73.5%	0.632	Synergism
2	25	25	70.2%	0.407	Synergism
3	12.5	12.5	66.8%	0.261	Strong Synergism
4	6.25	6.25	56.5%	0.265	Strong Synergism
5	3.125	3.125	50.0%	0.206	Strong Synergism
6	1.5625	1.5625	47.2%	0.054	Very Strong Synergism

E		NHF	Phenolic plus Docetaxel		
Points	Ph. Conc ug/ml	Doc. Conc. ug/ml	Cytotoxicity	CI	Effect
1	50	50	44.6%	0.346	Synergism
2	25	25	38.1%	0.307	Strong Synergism
3	12.5	12.5	34.1%	0.222	Strong Synergism
4	6.25	6.25	30.9%	0.152	Strong Synergism
5	3.125	3.125	28.7%	0.095	Very Strong Synergism
6	1.5625	1.5625	25.0%	0.071	Very Strong Synergism

F		NHF	Terpene plus Docetaxel		
Points	Ter Conc ug/ml	Doc. Conc. ug/ml	Cytotoxicity	CI	Effect
1	50	50	46.4%	0.277	Strong Synergism
2	25	25	42.5%	0.187	Strong Synergism
3	12.5	12.5	22.0%	0.622	Synergism
4	6.25	6.25	19.8%	0.407	Synergism
5	3.125	3.125	8.1%	1.724	Antagonism
6	1.5625	1.5625	6.0%	1.708	Antagonism

CompuSyn ^®^ was used to determine combination index. (A, B) show combination index (CI) values on the AMJ13 cancer cell line of the combined phenolic fraction with docetaxel and the terpene fraction with docetaxel respectively. (C, D) Represent the CI values of the phenolic fraction with docetaxel and the terpene fraction with docetaxel respectively, on the SK-GT-4 cancer cell line. (E, F) CI values of the phenolic fraction with docetaxel and the terpene fraction with docetaxel respectively, on the NHF non-cancer cell line. The effects have been described according to Hernández *et al.*, 2013. ^42^

As seen in the current study and after crystal violet staining, the AMJ13, SK-GT-4, and NHF cells that were exposed to 72h of extracted fractions and docetaxel and the combination therapy revealed cell shrinkage, cytoplasm and cell membrane disappearance, stromal edema, nucleus shrinkage and a marked decrease in the number of cells compared with control (untreated) cells. The effect of combination therapy was more prominent than single therapy as the cells showed more shrinkage, extensive cell damage, and necrosis.

As a result of their specificity, chemopreventive medicines only target cancer cells. A compound’s ability to selectively destroy cancer cells while having a minimal effect on healthy cells is measured by its “selectivity index.”

The compound’s low selectivity index suggests that it is less hazardous to healthy cells than cancerous ones. Compounds having high SI values may provide a safer and more effective cancer treatment option. ^48^

Based on the test results the phenolic fraction is found to be less selective for all tested cells (SI<3). Meanwhile, terpene fraction is selective for AMJ13 cancer cells with (SI>3) and less selective for SK-GT-4 cancer cells. The Docetaxel treatment (positive control) showed an excellent SI toward SK-GT-4 cell line (SI>3). The results of this study need a further evaluation to determine the potential cytotoxicity in animal models.

## Conclusions

It was observed from the results that the *P. arabica* phenolic and terpene extracts have significant cytotoxic activity on breast cancer and esophageal cancer cell lines with minimal effect against normal cells, due to the presence of effective compounds in this extract. Moreover, these active compounds increased the cytotoxic activity of docetaxel on cancer cell lines without increasing the toxicity against normal cells. Previous studies proved the cytotoxic effect of phenolic and phytosterol fractions extracted from other *Prunus* species. It is recommended to use a variety of cancer cell lines and animal models to evaluate the efficacy and safety of plant extracts, investigate their mechanisms of action, and explore their potential as therapeutic agents for developing novel cancer drugs from natural sources.

## Data Availability

Zenodo: Terpene Fractions Extracted from Iraqi *Prunus arabica* against AMJ13 and SK-GT-4 Human Cancer Cell Lines, https://doi.org/10.5281/zenodo.7618326. ^49^ The project contains the following underlying data:
•
Figure 1 - percent of cytotoxicity AMJ13 cell line.csv (the table illustrates the percent of cytotoxicity of serial concentrations of each fraction alone and combined with docetaxel for AMJ13 cancer cell line) •
Figure 2 - percent of cytotoxicity SK-GT-4 cell line.csv •
Figure 3 - percent of cytotoxicity - NHF cell line.csv (the table illustrates the percent of cytotoxicity of serial concentrations of each fraction alone and combined with docetaxel for NHF cell line) •
Figure 4 - A - IC 50 Docetaxel- AMJ13 cell line.csv (Cell viability values for serial concentrations of docetaxel in triplicate. Docetaxel exhibited cytotoxicity against AMJ13 cancer cell line with IC50 values of 14.51±0.77 μg/ml) •
Figure 4 - A - IC 50 Phenolic fraction-AMJ13 cell line.csv (Cell viability values for serial concentrations of phenolic fraction in triplicate. Phenolic fraction exhibited cytotoxicity against AMJ13 cancer cell line with IC50 values of 29.34±2.37 μg/ml) •
Figure 4 - A - IC 50 Terpene fraction-AMJ13 cell line.csv (Cell viability values for serial concentrations of terpene fraction in triplicate. Terpene fraction exhibited cytotoxicity against AMJ13 cancer cell line with IC50 values of 8.455±3.022 μg/ml) •
Figure 4 - B - IC50 Docetaxel- SK-GT-4 cell line.csv (Cell viability values for serial concentrations of docetaxel in triplicate. Docetaxel exhibited cytotoxicity against SK-GT-4 cancer cell line with IC50 values of 0.7125±0.084 μg/ml) •
Figure 4 - B - IC50 Phenolic fraction-SK-GT-4 cell line.csv (Cell viability values for serial concentrations of phenolic fraction in triplicate. Phenolic fraction exhibited cytotoxicity against SK-GT-4 cancer cell line with IC50 values of 21.97±3.56 μg/ml) •
Figure 4 - B - IC50 Terpene fraction-SK-GT-4 cell line.csv (Cell viability values for serial concentrations of terpene fraction in triplicate. Terpene fraction exhibited cytotoxicity against SK-GT-4 cancer cell line with IC50 values of 15.14±3.266 μg/ml) •
Figure 4 - C - Docetaxel- IC50 NHF cell line.csv (Cell viability values for serial concentrations of docetaxel in triplicate. Docetaxel exhibited cytotoxicity against NHF cell line with IC50 values of 24.9±7.17 μg/ml) •
Figure 4 - C - phenolic fraction- IC50 NHF cell line.csv (Cell viability values for serial concentrations of phenolic fraction in triplicate. Phenolic fraction exhibited cytotoxicity against NHF cell line with IC50 values of 18.07±1.83 μg/ml) •
Figure 4 - C - Terpene fraction- IC50 NHF cell line.csv (Cell viability values for serial concentrations of terpene fraction in triplicate. Terpene fraction exhibited cytotoxicity against NHF cell line with IC50 values of 31.81±12.07 μg/ml) •
Figure 5 - A - Phenolic plus docetaxel- AMJ13.html (Cytotoxicity of *Prunus arabica* phenolic extract combination with docetaxel against AMJ13 cancer cell line. I-Dose-Effect Curve at 50% cytotoxicity, II-Isobologram analysis displays synergism rate between phenolic fraction and docetaxel at all points of the combination as they located at the lower left of the hypotenuse, demonstrating the effect is synergistic at a 50% cytotoxicity dose, III-showing the combination index data location for each dose.) •
Figure 5 - C - Phenolic plus docetaxel SK-GT-4.html (Cytotoxicity of *Prunus arabica* phenolic extract combination with docetaxel against SK-GT-4 cancer cell line. I-Dose-Effect Curve at 50% cytotoxicity, II-Isobologram analysis displays synergism rate between phenolic fraction and docetaxel at all points of the combination as they located at the lower left of the hypotenuse, demonstrating the effect is synergistic at a 50% cytotoxicity dose, III-showing the combination index data location for each dose.) •
Figure 5 - F - Terpene plus docetaxel NHF.html (Checking the possible combination cytotoxicity against NHF cell line. There is no cytotoxic effect for terpene fraction against NHF cell line in combination with docetaxel on cell viability. I-Dose–response curve at shows response less than 50%, II-Isobologram analysis showing no synergism between terpene fraction and docetaxel against NHF at all doses tested, III-The figure of combination index showed the absence of any synergistic points.) •
Figure 5 - E - Phenolic plus docetaxel NHF.html (Checking the possible combination cytotoxicity against NHF cell line. There is no cytotoxic effect for phenolic fraction against NHF cell line in combination with docetaxel on cell viability. I-Dose–response curve at shows response less than 50%, II-Isobologram analysis showing no synergism between terpene fraction and docetaxel against NHF at all doses tested, III-The figure of combination index showed the absence of any synergistic points.) •
Figure 5 - B - Terpene plus docetaxel AMJ13.html (Cytotoxicity of *Prunus arabica* terpene fraction combination with docetaxel against AMJ13 cancer cell line. I-Dose-Effect Curve at 50% cytotoxicity, II-Isobologram analysis displays synergism rate between terpene fraction and docetaxel at all points of the combination as they located at the lower left of the hypotenuse, demonstrating the effect is synergistic at a 50% cytotoxicity dose, III-showing the combination index data location for each dose.) •
Figure 5 - D - Terpene plus docetaxel SK-GT-4.html (Cytotoxicity of *Prunus arabica* terpene fraction combination with docetaxel against SK-GT-4 cancer cell line. I-Dose-Effect Curve at 50% cytotoxicity, II-Isobologram analysis displays synergism rate between terpene fraction and docetaxel at all points of the combination as they located at the lower left of the hypotenuse, demonstrating the effect is synergistic at a 50% cytotoxicity dose, III-showing the combination index data location for each dose.) •
Table 2 - HPLC phenolic fraction.pdf (The table illustrates the composition of phenolic fraction, retention time and their concentrations) •
Table 3 - HPLC Sterol fraction.pdf (The table illustrates the composition of terpene fraction, retention time and their concentrations) Data are available under the terms of the Creative Commons Attribution 4.0 International license (CC-BY 4.0). 2- protocols.io: A full step by step assay protocols Plant Extraction and Fractionation, https://dx.doi.org/10.17504/protocols.io.q26g7y6bqgwz/v1. ^50^ Preliminary qualitative phytochemical analysis, https://dx.doi.org/10.17504/protocols.io.e6nvwj27wlmk/v1. ^51^ MTT (Assay protocol), https://dx.doi.org/10.17504/protocols.io.eq2ly72emlx9/v1. ^52^ Data are available under the terms of the Creative Commons Attribution 4.0 International license (CC-BY 4.0).
