# Supplementary material for: Assessing the cytotoxicity of phenolic and terpene fractions extracted from Iraqi Prunus arabica against AMJ13 and SK-GT-4 human cancer cell lines
Source: F1000Res. 2024 Sep 10;12:433. Originally published 2023 Apr 21. [Version 3] doi: 10.12688/f1000research.131336.3 (PMC11480737; doi:10.12688/f1000research.131336.3)
Supplement: Peer review history

# Reviewer report: Reviewer response for version 3

**DOI:** 10.5256/f1000research.171204.r327511

**Published:** 2024-10-15

**Authors:** Purwantiningsih Sugita

**Affiliations:** 1 IPB University, Dramaga, Bogor, Indonesia

This article discusses the effects of Iraqi Prunus arabica phenolic and terpene fractions on the human cancer cell lines SK-GT-4 and AMJ13 **. The content is well-written and and has accommodated feedback for improvements from reviewers. **I suggest this manuscript can proceeded to next step in this journal. Thank you for invite me as reviewer.

Is the work clearly and accurately presented and does it cite the current literature?

Yes

If applicable, is the statistical analysis and its interpretation appropriate?

Yes

Are all the source data underlying the results available to ensure full reproducibility?

Yes

Is the study design appropriate and is the work technically sound?

Yes

Are the conclusions drawn adequately supported by the results?

Yes

Are sufficient details of methods and analysis provided to allow replication by others?

Yes

Reviewer Expertise:

Natural organic chemistry; Biomaterial

I confirm that I have read this submission and believe that I have an appropriate level of expertise to confirm that it is of an acceptable scientific standard.

# Reviewer report: Reviewer response for version 2

**DOI:** 10.5256/f1000research.150979.r281243

**Published:** 2024-06-04

**Authors:** Heba A. S. El-Nashar

**Affiliations:** 1 Ain Shams University, Cairo, Egypt

This article describes the cytotoxicity of phenolic and terpene fractions extracted from Iraqi Prunus arabica on AMJ13 and SK-GT-4 human cancer cell lines. The article is well-written, some points need to be thoroughly revised to be considered for publication. The following are some comments to be considered:

1. Title... please change “on” to “against”.
2. Abstract….Please, Remove the following word: According to the findings, as conclusion
3. The following statement” The study aimed to evaluate the cytotoxicity of phenolic and terpene fractions extracted from Iraqi Prunus arabica on breast (AMJ13) and esophageal (SK-GT-4) cancer cell lines by using the MTT assay (3-(4,5-dimethylthiazol-2-yl)-2,5-diphenyl-2H-tetrazolium bromide).” Should be transferred to the background.
4. Prunus arabica should be written in italic, Please Check throughout the whole manuscript.
5. Keyword should be written in alphabetical order.
6. Introduction--Some sentences lack citations, please Check them thoroughly.
7. Introduction & Discussion--Please consider the recent citations in the field of cytotoxic plant extracts as follows; https://doi.org/10.1080/14786419.2020.1765343; https://doi.org/10.1186/s12906-023-04164-9; https://doi.org/10.1016/j.jpba.2024.116121; https://doi.org/10.1002/cbdv.202300847.
8. Materials and methods…Please, define which part of the plant did you use?
9. Materials and methods- Please, Acknowledge the taxonomist who identify this plant and his affiliation.
10. Please, Mention the voucher sample of the plant and its code.
11. Discussion.…You should correlate the cytotoxic activity with the identified compounds of the extract.
12. Conclusion part… It is important to mention the limitations and the future perspectives regarding this study.
13. Some grammatical errors should be corrected throughout the whole manuscript.

Is the work clearly and accurately presented and does it cite the current literature?

Partly

If applicable, is the statistical analysis and its interpretation appropriate?

Yes

Are all the source data underlying the results available to ensure full reproducibility?

Yes

Is the study design appropriate and is the work technically sound?

Yes

Are the conclusions drawn adequately supported by the results?

Partly

Are sufficient details of methods and analysis provided to allow replication by others?

Partly

Reviewer Expertise:

Plant science and phytochemistry

I confirm that I have read this submission and believe that I have an appropriate level of expertise to confirm that it is of an acceptable scientific standard, however I have significant reservations, as outlined above.

# Reviewer report: Reviewer response for version 2

**DOI:** 10.5256/f1000research.150979.r190235

**Published:** 2023-08-29

**Authors:** Serap Sahin-Bolukbasi

**Affiliations:** 1 Department of Biochemistry, Faculty of Pharmacy, Afyonkarahisar Health Sciences University, Afyonkarahisar, Turkey

The authors have revised the manuscript and the overall quality has been improved. Hence, I recommend the acceptance of the article without further changes.

Is the work clearly and accurately presented and does it cite the current literature?

Yes

If applicable, is the statistical analysis and its interpretation appropriate?

Yes

Are all the source data underlying the results available to ensure full reproducibility?

Yes

Is the study design appropriate and is the work technically sound?

Yes

Are the conclusions drawn adequately supported by the results?

No

Are sufficient details of methods and analysis provided to allow replication by others?

Yes

Reviewer Expertise:

Lipidomics, Cell culture, Cytotoxic activity tests, Biotechnological drug design,

I confirm that I have read this submission and believe that I have an appropriate level of expertise to confirm that it is of an acceptable scientific standard.

# Reviewer report: Reviewer response for version 1

**DOI:** 10.5256/f1000research.144170.r171852

**Published:** 2023-05-23

**Authors:** Serap Sahin-Bolukbasi

**Affiliations:** 1 Department of Biochemistry, Faculty of Pharmacy, Afyonkarahisar Health Sciences University, Afyonkarahisar, Turkey

This manuscript presents high-performance liquid chromatography (HPLC) analysis of quantitative determination of different bioactive molecules of both phenolic and terpen fractions in the Iraqi *Prunus arabica* extract. The researchers evaluated the synergistic effect between the extracted fractions and chemotherapeutic agent (docetaxel). The cytotoxic activity of phenolic and terpene fractions extracted from Iraqi *Prunus arabica* against breast (AMJ13) and esophageal (SK-GT-4) cancer cell and also normal human fibroblasts (NHF) cell.

 However, the manuscript needs to be improved in order to meet the qualifications that are required for indexing. There are some issues that should be checked and corrected before indexing:

1. Please use subscript for IC _50_ (whole manuscript).
2. Please check whole manuscript for gm (it should be g?).
3. Please write which solvent was used to control wells and what was the concentration of solvent in control wells?
4. Please add ±SD values of samples in each concentration in Figure 1-3.
5. Please add ±SD values of samples in each IC _50 _concentration in manuscript.
6. Morphological analysis (crystal violet staining) section, I would like to see images of IC _50_ of the phenolic fraction, terpene, docetaxel, and their combinations with chemotherapy.
7. Please write the source of AMJ13, SK-GT-4 and NHF cell line (ATCC, ECACC, Sigma-Aldrich or other…), even if it's a gift.
8. Page 7; ‘...while a CI value larger than 1.1, the effects were assumed to be antagonistic...’ and Figure 5 legends page 9; ‘...CI value less than 1 indicates an antagonism...’’ CI value less than or larger tahan 1 assumed antagonistic? Please correct.
9. Please add ‘half’ in Figure 4 legend; ‘Figure 4. The **half** maximal inhibition concentration (IC _50_) for phenolic, terpene, and docetaxel, respectively, on A: AMJ13, B: SK-GT-4, and C: NHF cell lines.’
10. Please write axis name in Figure 4.
11. The selectivity index is an important parameter for indicates the selectivity of a given samples between normal and cancer cells. Samples are expected less cytotoxic towards the healthy cells according to the cancer cells. Please calculate the selectivity indexes of samples.
12. Authors should compare the results of this study some other similar plant extracts with more details. References should be improved and updated in the results and discussion section. Indeed, the field of similar samples with regard to both their chemistry and biological relevance, include a significant number of articles and some recent reviews
13. Please use only one of the terms antiproliferative activity, cytotoxic activity, or anticancer activity in all manuscripts.

Is the work clearly and accurately presented and does it cite the current literature?

Yes

If applicable, is the statistical analysis and its interpretation appropriate?

Yes

Are all the source data underlying the results available to ensure full reproducibility?

Yes

Is the study design appropriate and is the work technically sound?

Yes

Are the conclusions drawn adequately supported by the results?

No

Are sufficient details of methods and analysis provided to allow replication by others?

Yes

Reviewer Expertise:

Lipidomics, Cell culture, Cytotoxic activity tests, Biotechnological drug design,

I confirm that I have read this submission and believe that I have an appropriate level of expertise to confirm that it is of an acceptable scientific standard, however I have significant reservations, as outlined above.

# Response

**Published:** 2023-06-05

**Authors:** matin mahmood

**Affiliations:** alkitab university, Iraq

Dear Assoc. Prof. SERAP ŞAHİN BÖLÜKBAŞI,

 Thank you for your thoughtful review of our manuscript. We greatly appreciate your feedback and suggestions for improvement. In our experiments, we used untreated cells, MTT solution, and a solubilizing buffer (DMSO) as controls. To ensure the integrity of our experimental results and minimize any potential confounding effects, we carefully selected a final concentration of 0.5% DMSO for our study. This concentration has been widely reported in the literature as non-toxic and compatible with various cell types. Regarding the morphological analysis section of this study, it is worth noting that the analysis was carried out using only a concentration of 100 ug/ml, and it is important to clarify that no analysis was conducted specifically to IC _50 _values.

 Sincerely, Matin A. Mahmood
